# Editorial: Women in mental health services: volume 2

**DOI:** 10.3389/frhs.2025.1702469

**Published:** 2025-10-22

**Authors:** Theresa Van Lith, Kristen A. Morin

**Affiliations:** ^1^ Department of Psychology, Counselling and Therapy, School of Psychology and Public Health, La Trobe University, Melbourne, VIC, Australia; ^2^ Northern Ontario School of Medicine, Thunder Bay, ON, Canada

**Keywords:** women, mental health, cultural resonance, sustainability, relational interventions

**Editorial on the Research Topic**
Women in mental health services: volume 2

Women's mental health emerges at the intersection of clinical realities, cultural contexts, and social and structural influences. This second volume of *Women in Mental Health Services* brings together research that illuminates these layers of complexity while pointing to new ways of understanding and supporting women in diverse contexts. Rather than presenting final answers, the contributions in this collection invite critical reflection on paradoxes, reconsideration of established practices, and thoughtful exploration of how services might evolve in response to women's lived experiences.

A central thread running through the volume is the perspective that women themselves bring to mental health services. Women's voices shape the research questions asked, highlight gaps in existing service models, and broaden what is considered effective care. Whether through participation in research, leadership in service design, or experiential accounts, women's perspectives anchor these studies in lived reality. This perspective ensures that innovations are not only responsive to systemic pressures but are grounded in the needs, identities, and strengths of those most affected.

The COVID-19 pandemic revealed both the fragility and adaptability of mental health systems, exposing how crises can disrupt access while also prompting innovation. Russolillo et al. examined inpatient psychiatric admissions in Vancouver, documenting shorter lengths of stay for some diagnostic categories, such as anxiety and personality disorders, during the pandemic. These patterns may reflect systemic pressures or changing priorities in acute care. At the same time, the persistence of admissions for severe conditions, including schizophrenia, underscored that inpatient services remained indispensable even under strain. Lemoine et al. extended this systems-level perspective by analyzing visits to a 24-hour walk-in crisis centre in Winnipeg. They found that service utilization remained lower than expected, even as broader indicators suggested increasing public need. Together, these studies show that demand for mental health support does not necessarily translate into service use, with stigma, fear of contagion, and structural barriers shaping whether and how women seek help during times of crisis.

Attention to cultural context adds further depth to this volume. Marsh et al. examined the experiences of women participating in an Indigenous residential treatment program in Northern Ontario. Participants described the healing value of traditional practices and the Medicine Wheel's holistic framework, which supported interconnected spiritual, emotional, physical, and community dimensions of recovery. Yet the program's abstinence-based model excluded women using opioid agonist therapies, revealing tensions between cultural grounding and biomedical approaches. These findings remind us that culturally meaningful care can be both empowering and constraining, and they highlight the ongoing challenge of designing programs that respect Indigenous sovereignty while accommodating diverse treatment needs.

Reproductive and sexual health services represent another critical domain for advancing women's mental health. Khosropour et al. investigated the integration of HIV pre-exposure prophylaxis (PrEP) into OB/GYN practices in upstate New York. Their findings revealed gaps in both patient and provider awareness: some women perceived PrEP as relevant only for other populations, while providers expressed uncertainty about prescribing it. Despite these barriers, both groups recognized the importance of broadening access. The study points to the opportunities and challenges of embedding preventive strategies within reproductive care, underscoring the need for structural and cultural shifts to normalize comprehensive approaches to women's health. Parallel work in South Africa has demonstrated that embedding mental health interventions into PrEP services for adolescent girls and young women can enhance uptake and adherence, particularly when implemented through human-centered approaches (1).

Creative and relational interventions also feature prominently in this volume. Han et al. reported on *Melodies for Mums*, a singing-based program for women experiencing postnatal depression. Their mixed-methods evaluation showed that group singing supported not only reductions in depressive symptoms but also strengthened solidarity, restored a sense of identity, and fostered agency among participants. By identifying the “active ingredients” that shaped feasibility and acceptability, such as social connection and shared expression, the study demonstrates how creative practices can provide meaningful pathways to recovery. The findings also align with broader evidence on the role of arts and cultural engagement in promoting health and wellbeing (2), highlighting how non-traditional interventions can complement biomedical and psychological approaches.

Concurrently, these contributions illustrate the intricacies of women's mental health and the necessity of nuanced, multi-layered approaches. Some of the articles reveal how health systems become strained and less accessible during times of crisis. Others emphasize the importance of cultural grounding, even as they confront tensions between traditional and biomedical frameworks. Still others demonstrate how innovations in reproductive care and creative practice can expand the scope of what is considered effective support. What links them is a recognition that women's mental health is not defined by a single pathway but is shaped by diverse contexts, knowledge systems, and lived realities.

This volume also resonates with emerging evidence outside the special issue. For instance, recent economic evaluations suggest that even low-cost psychosocial interventions can significantly reduce stress and depression among women during crises (3). Such findings underscore that scalable, context-sensitive strategies have the potential to complement the more targeted innovations featured here.

*Women in Mental Health Services: Volume 2* encourages us to reconsider assumptions, to engage with paradoxes rather than avoid them, and to support innovation that grows from women's lived experiences. By highlighting the perspectives women bring to mental health services, shaping research agendas, broadening services, and expanding relational and cultural possibilities, the collection demonstrates that women are not only service users but also co-creators of knowledge and care. The challenge ahead is not only to continue documenting disparities but also to build research and practice that are anticipatory, adaptive, and collaborative. Future studies might explore how to embed greater flexibility into mental health systems so they can better withstand crises, how to integrate culturally grounded knowledge with biomedical approaches in ways that respect both, how to normalize preventive strategies such as PrEP within reproductive health, and how to expand creative and non-traditional interventions without losing their relational depth. By treating complexity as an invitation to inquiry rather than a barrier to progress, scholars and practitioners can help shape services that are not only clinically effective but also equitable, culturally resonant, and sustainable for the future.
